# Transactions of the Obstetrical and Gynæcological Section of the 43d Congress of the German Physicians and Physicists, at Insbruck, Sept. 18–24, 1869

**Published:** 1870-04

**Authors:** James T. Whittaker

**Affiliations:** Cincinnati, Ohio


### TRANSACTIONS OF THE OBSTETRICAL AND GYNÆCOLOGICAL SECTION OF THE 43D CONGRESS OF THE GERMAN PHYSICIANS AND PHYSICISTS, AT INSBRUCK, SEPT. 18–24, 1869.

BY JAMES T. WHITTAKER, Cincinnati, Ohio.

*Schmidts Jahrbucher*, Nov. 30, 1869.

Four sessions, under the Chairmanships of Prof. Lange, Heidelberg; Prof. Spiegelberg, Breslau; Dir. Birnbaum, Cologne; Prof. Späth, Vienna; respectively.

*Essays*—Prof. *Lange*, on a case of "Cæsarian Section, from a Pelvis with the Transverse Contraction," to be fully published in the *Monabschift f. Geburtskunde.*

Prof. *Spiegelberg*, on the "Value of the Induction of Premature Labor;" according to his own experience (305 cases in the clinic and polyclinic of Breslau), and according to that of others, the induction of premature labors does not afford the results which are usually expected, since it is not to be practiced in pelves of 3 in. conjugate and over, and is only admissible in greater contraction when previous labors have been characterized by large resisting heads, or unfavorable presentations, or when great injury might ensue to soft parts already in disease. The habitual occurrence of death in the two last months of pregnancy, presents no indication, since this accident is caused by hereditary syphilis, which the interruption of pregnancy does not destroy. The premature induction is only justifiable in disease of the mother, caused or aggravated by pregnancy, and only then when there is hope of rescue to the mother's life. As regards the termination of the birth, with or without induction, in answer to Prof. Dohrn, Sp. remarked that he had had unfortunate results in three cases, in the same patient. The reason that more favorable results occurred in private practice than in clinics, seemed to depend upon the more frequent examinations, by the students, in the latter case. Dr. *Birnbaum* had observed habitual death of the fœtus in chronic induration of the cervical mucous membrane, and had applied injection of cup. sulph., in the interval of two pregnancies, with success. Prof. *Hegar*, Freiberg, agreeing with Prof. Sp., in regard to premature delivery, had also noticed habitual death without syphilis, and had observed, after catheterism and injections into the uterus, inflammatory affections, even with fatal termination.

Prof. *Breisby*, Bern, "On the Condition of the Cervix during Labor." Physiological softening arises, with lengthening and paralysis, caused by the pains and by pressure of the membranes, as well as by the presenting parts. He had found, by measurement with the finger, a lengthening of even 6 ctmtr., and called attention to the importance of the cervical condition in originating flexions in the puerperal bed. Rupture of the cervix is often caused by physical peculiarities of its tissue.

Prof. *Freund*, on "Parametritis chronica atrophicans," an affection, in its anatomical sub-strata, but little known, from its symptoms, called hysteria. (To be published in full.)

Prof. *Dohrn*, Marbury, on the "Tubes of Müller and the Development of the Uterus." From the various examinations of human and animal embryos, the following conclusions are drawn: *a*, The coalition of the tubes of Müller begins between the middle and lower third of the genital cord, advances, then, upward and downward, but downward faster; this accounts for the fact that separation often exists in the upper portion of the female genital cord. *b*, The left tube lies higher, generally, than the right, and the coalition occurs in this oblique position. The pressure of the intestinal extremity, on the left, is the cause of this elevation, which thus explains the frequent version of the uterus on its axis, so that its left border is turned forward. *c*, In man, the coalition occurs early, being finished in its entire course by the end of the second month of embryo life.

Prof. *Spiegelberg*, on the "Diagnostic Significance of the Puncture of Ovarian Tumors." Of more importance in the differentiation of cysts of the ovary from those of other organs, of less for a distinction between the different kinds of ovarian cysts themselves. Of particular value is the exploratory puncture for the differential diagnosis of ovarian cysts from ascites; besides the greater quantity of albumen, and the multitudinous forms of epithelial structure, he denotes as characteristic for ovarian cysts the absence of the fibrogenous substance present in peritoneal exudation with the clot (gerinnsel) formations dependent thereon; as well as of the movable amœboid cells. He regards the tentative puncture as irremissible, before every attempt at radical cure, although it is not always so innocent as is generally believed, and in this he is corroborated by Prof. *Hegar*, who has seen the discharge continue, and a blood effusion occur in the emptied space, with fatal termination, and this from a puncture of the cyst, through the vagina, with a very fine trocar.

Prof. *Kehrer*, Giessen, demonstrated the "Mechanism of several Pelvic Deformities," on two caoutchouc pelves, decalcinated with nitric acid. The form of the flat rachitic pelvis is caused by the weight of the body, and the counter-pressure of the femora, upward traction of the spinal extensors on the extremity of the sacrum (with diminished action of the abdominal muscles), and traction of the rotators on the coccyx. The osteo-malacic pelvis originates, besides from pressure and counter-pressure of the body and femora, from relaxation of the spinal extensors and the quadratic lumborum, with secondary increase of their antagonists, the muscles of the abdomen, the median fasiculus, and the illiaci. The oblique oval pelvis is due to the fact that the weight of the body is supported in the direction of the distantia sacro-condyloidea, the cyphotic oblique contraction, by upward and downward pressure, in the direction of the base of the sacrum. According to Dr. *Freund*, Breslau, the sacrum is thinner in the rachitic pelvis, and longer anteriorly than posteriorly, because the burden of the body is not transmitted through the bodies of the vertebræ, but through their oblique processes. *Kehrer* explains the convex form of the anterior sacral surface in the rachitic pelvis by the diminished firmness of union of the intervertebral discs, permitting a version of the sacrum and a change of form, so that its angle is directed backward. The great flattening of the anterior wall of the pelvis in rachitis, as compared to the beak form of osteomalacia, finds an explanation in the fact that the softening process in the former affects to a greater degree the extremities of the bones, while in the latter, the process is more universal. Prof. *Hegar* considers the traction of the muscles as less effective because it implies a greater (not demonstrable) activity of their contractile power. *Kehrer* explains the symmetrical traction of the spinal muscles, by the coincident relaxation of the abdominal muscles in the rachitic child. *Birnbaum* saw a case of the oblique contraction caused by tight lacing.

Dr. *Schatz*, Leipzig, on the "Mechanism of Comparative Labor." (To be published in full.)

*M. R. Kuchenmeister* presented several gynæcological instruments. (*a.*) A pair of scissors, with a projection at right angles on one of the blades, to gauge the extent of the section of the portio vaginalis, and to avoid making it shorter than designed. Prof. Speigelberg found the action of the scissors certain, but they caused often a severe hemorrhage, by tearing the tissue, a hemorrhage which might render the application of cauterization necessary, and this leads to parametritis. K. believed that tearing might be avoided by a careful section. (*b.*) A curved trocar, for the puncture of hematocle, which might also arise from an escape of blood from the accessory tubes often (?)* found, or from the orifices of the Fallopian tubes during menstruation.

Prof. *Kehrer* maintained that an effusion of blood could only originate from the Graafian follicle, or, *pathologically*, from the mucous membrane of the tube; an escape from the uterus through the tubes is very improbable, on account of the slow discharge of the fluid. Prof. Freund inserts a short trocar, thicker at its upper extremity, and lengthened by a catheter, and leaves it there. (*c.*) A forceps for the removal of polypi out of reach of the finger.

Prof. *Spiegelberg* on "Galvano-caustic Operations on the Uterus." Amputation of the collum uteri, with the knife, especially for carcinoma, is followed by very severe hemorrhage, with the ecraseur, hemorrhage is generally, though not by any means always, prevented, but the neighboring parts suffer from pressure. The disadvantages mentioned are avoided by the galvano-caustic noose. The wire must be applied as nearly as possible to the vaginal insertion,† and to effect this, a spatula-shaped instrument will prove of service. The noose is to slowly attain a white heat, and to be allowed to slowly cut its way through, whereby it leaves a smooth surface of good granulation. The operation induces no hemorrhage of mention, and is not painful, indeed the heat will not be even perceived if cold water be played upon the field of operation. In carcinoma, the operation is only palliative, as return has always been observed.

Regarding intra-uterine galvano-caustic treatment, Sp. re-

* According to the observations of Richard, besides the regular orifice, there occur in certain cases other lateral openings, also provided with fimbræ. He found them five times in thirty cases, and either in the neighborhood of the proper orifices, or in the middle of the tube. In one case, the lateral opening conducted into a short membranous tube, communicating with the Fallopian. I have lately discovered such a lateral orifice in immediate proximity to the proper *ostium abdominalæ tubæ.—Hyrtl Anatomie des Menschen.*—[*Trans.*

† Another decided advantage of the galvano-caustic noose is the comparative impunity with which a portion of the peritonium may be removed, where the disease is so extensive as to involve the supra-vaginal cervix. We can recall a case in Prof. Braun's clinic, at Vienna, wherein a full square inch was abstracted, and the patient recovered.—[*Trans.*

marked that he was opposed to intra-uterine treatment in general. In any case, dilation must first be effected. This is best done with the sponge tent. He had seen metritis and parametritis follow the injection of alkalis and iodine, more seldom, however, the use of argent. nit., and other astringents of more superficial action. In blenorrhagia and hemorrhages from endometritis polyposa, he had cauterized the uterine cavity with a porcelain burner, curved like the uterine sound, but he had always interrupted the current as soon as the patient complained of pain In no case had he observed any great reaction. For the first few days after the operation, no discharge existed; later, profuse and mixed with blood; this, however, ceased entirely within fourteen days. Prof. *Lange* and *Hegar* confirmed Sp.'s experience regarding the ecraseur in cervical amputation. *Hegar* prefers the knife and scissors, as easier manipulated, and as accelerating cure, and advises that the incision be made oblique and funnel-shaped (trichterförmig). To quell the hemorrhage he employs the suture of Sims, uniting the mucous membrane to the vagina at its insertion; further compression is not necessary. *Spiegelberg* considers the funnel incision with the suture as adaptable in simple elongations, but not in severe carcinomatous degeneration, where recurrence is to be feared. *Hegar* responds that he first removes the main mass, and then carries the incision into the cervix; the suture certainly does not effect union in carcinoma by prima intentio, but it is a good hemostatic, and renders compression and tampon superfluous. Prof. *Spath* confirms, in general, the disadvantages of the intra-uterine treatment of flexions; he agrees with Sims, that the uterus bears the bloody better than other procedures; the unfortunate results with the laminaria remaining unpublished. Dr. *Zini*, Graz, had seen two cases of unfavorable termination with the laminaria, and Dr. *Hugenberger*, Petersburgh, had observed a pyæmic condition result in one case, after its use. In flexions, he employs the sound of Simpson, which is well borne, when of proper length, and properly applied.—*Lancet and Observer.*

